# Identification of ADAR1 adenosine deaminase dependency in a subset of cancer cells

**DOI:** 10.1038/s41467-018-07824-4

**Published:** 2018-12-21

**Authors:** Hugh S. Gannon, Tao Zou, Michael K. Kiessling, Galen F. Gao, Diana Cai, Peter S. Choi, Alexandru P. Ivan, Ilana Buchumenski, Ashton C. Berger, Jonathan T. Goldstein, Andrew D. Cherniack, Francisca Vazquez, Aviad Tsherniak, Erez Y. Levanon, William C. Hahn, Matthew Meyerson

**Affiliations:** 1 Department of Medical Oncology, Dana-Farber Cancer Institute, Boston, MA 02215 USA; 2 Broad Institute of Harvard and MIT, Cambridge, MA 02142 USA; 3 Department of Gastroenterology and Hepatology, University of Zurich and University Hospital Zürich, Zürich, 8091 Switzerland; 4 Department of Medical Oncology and Hematology, University Hospital Zurich, University of Zurich, 8091 Zurich, Switzerland; 5 The Mina and Everard Goodman Faculty of Life Sciences, Bar-Ilan University, 52900 Ramat Gan, Israel; 6 Department of Medicine, Brigham and Women’s Hospital, Harvard Medical School, Boston, MA 02115 USA; 7 Center for Cancer Genome Discovery, Dana-Farber Cancer Institute, Boston, MA 02215 USA; 8 Department of Pathology, Harvard Medical School, Boston, MA 02115 USA

**Keywords:** Lung cancer, Tumour immunology

## Abstract

Systematic exploration of cancer cell vulnerabilities can inform the development of novel cancer therapeutics. Here, through analysis of genome-scale loss-of-function datasets, we identify adenosine deaminase acting on RNA (*ADAR* or ADAR1) as an essential gene for the survival of a subset of cancer cell lines. ADAR1-dependent cell lines display increased expression of interferon-stimulated genes. Activation of type I interferon signaling in the context of ADAR1 deficiency can induce cell lethality in non-ADAR1-dependent cell lines. *ADAR* deletion causes activation of the double-stranded RNA sensor, protein kinase R (PKR). Disruption of PKR signaling, through inactivation of PKR or overexpression of either a wildtype or catalytically inactive mutant version of the p150 isoform of ADAR1, partially rescues cell lethality after ADAR1 loss, suggesting that both catalytic and non-enzymatic functions of ADAR1 may contribute to preventing PKR-mediated cell lethality. Together, these data nominate ADAR1 as a potential therapeutic target in a subset of cancers.

## Abstract (web summary)

Specific cancer cell vulnerabilities can provide an opportunity for the development of novel cancer therapeutics. In this study the authors demonstrate that targeting ADAR1 represents a potential therapeutic vulnerability in cancers with activated interferon response signatures.

## Introduction

Despite the discovery and widespread use of novel targeted therapies that inhibit the activity of mutant oncogene products, such as EGFR and ALK^1,2^, and immunotherapies that modulate anti-tumor immunity^3–6^, lung cancer remains the leading cause of cancer death worldwide. Importantly, most lung cancer patients are not eligible for targeted therapies because their tumors lack a targetable genomic alteration. Moreover, a substantial proportion of lung cancer patients treated with immune checkpoint inhibitors do not achieve an objective response^4–6^. Thus, the discovery of novel therapeutic modalities remains critical to improving outcomes in lung cancer care.

Lung cancer cells may harbor specific genomic or functional alterations that render them vulnerable to particular genetic perturbations^7,8^. Identification of these synthetic lethal interactions may offer an opportunity for the development of novel classes of therapies for lung cancer. In this study, we utilize genome-scale loss-of-function datasets to uncover genetic dependencies in lung cancer cell lines. We find that lung cancer cell lines expressing high levels of interferon-stimulated genes (ISGs) are vulnerable to deletion of the RNA adenosine deaminase, *ADAR* or ADAR1. *ADAR* deletion induces phosphorylation of the cytoplasmic double-stranded RNA (dsRNA) sensor PKR, leading to downstream signaling. Deletion of PKR can partially rescue cell lethality after ADAR1 loss, indicating that *ADAR* genetic dependency is at least partly mediated by PKR signaling. Overexpression studies demonstrate that both the catalytic and non-enzymatic functions of ADAR1 may restrain PKR-mediated cell lethality in ADAR1-dependent lung cancer cell lines. Taken together, our data suggest that ADAR1 may represent a potential therapeutic target in cancers displaying activation of interferon response pathways.

## Results

### ADAR1 dependency in cancer cell lines with elevated ISGs

**Fig. 1:**
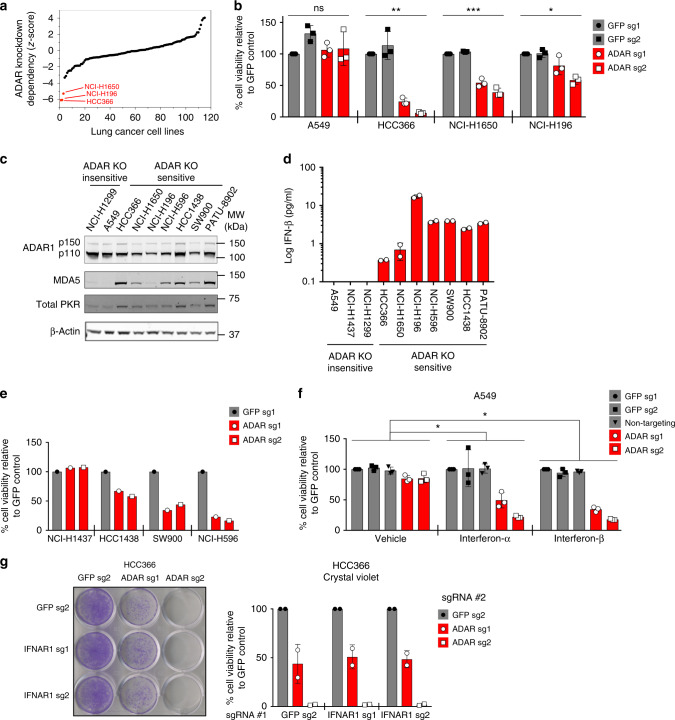
High expression of ISGs in cancer cell lines is predictive of sensitivity to *ADAR* deletion. **a***Z*-scores representing the degree of cell lethality after *ADAR* knockdown in lung cancer cell lines included in published genome-scale loss-of-function screens^9^. *Z*-scores represent the number of standard deviations from the mean for each data point. **b** Cell viability was assessed by ATP bioluminescence 11 days after *GFP* or *ADAR* KO with CRISPR-Cas9. ATP bioluminescence values were normalized to the GFP sg1 control within each cell line. Three independent biological replicates were performed for each cell line. *p = 0.0054, **p = 0.0008, and ***p < 0.0001 as calculated by the Kruskal–Wallis test. **c** Immunoblots showing protein levels of ISGs and β-actin (loading control) in *ADAR* KO-sensitive and KO-insensitive cancer cell lines (*n* = 3). **d** Spontaneous IFN-β secretion by *ADAR* KO-sensitive and KO-insensitive cancer cell lines as measured by ELISA 24 h after replacement of culture media. Technical replicates from one representative experiment are shown (*n* = 2). **e** Cell viability was assessed by ATP bioluminescence 11 days after *GFP* or *ADAR* KO with CRISPR-Cas9 in additional lung cancer cell lines. ATP bioluminescence values were normalized to the GFP sg1 control within each cell line (*n* = 1). **f** Cell viability of control or ADAR1-deficient A549 cells was assessed by ATP bioluminescence 3 days after vehicle or IFN-I treatment (10 ng/mL). ATP bioluminescence values were normalized to the GFP sg1 control within each treatment group. Three independent biological replicates were performed. Two-way ANOVA showed a significant interaction between *ADAR* KO and IFN-I treatment (*p < 0.0001, degrees of freedom = 8, F-ratio = 10.51). Dunnett’s multiple comparisons post-test showed a significant difference between vehicle and IFN-I treatment groups and between control and *ADAR* KO groups (*p < 0.0001). **g** Cell viability of control or IFNAR1-deficient HCC366 cells was assessed by crystal violet staining 11–13 days after *GFP* or *ADAR* KO with CRISPR-Cas9. A representative image of crystal violet staining (left) and quantitation of cell viability (right) from two independent biological replicates are shown. Cell viability values were normalized to the GFP sg2 control #2 within each group of isogenic cells. Error bars represent standard deviation in all graphs

We analyzed publicly available, genome-scale shRNA screening datasets^9^ in search of novel genetic dependencies in lung cancer. Based on previously described criteria^9^, we identified 11 genes that are potentially required for the survival of subsets of lung cancer cell lines (Supplementary Table 1). These genes included *SMARCA2* and *PRKDC*^7,8^, which have been shown to be synthetic lethal targets in subsets of lung cancers, as well as the adenosine deaminase acting on RNA gene, *ADAR*. Suppression of *ADAR* gene expression showed outlier lethality in HCC366, NCI-H196, and NCI-H1650 lung cancer cells compared to other tested lung cancer cell lines (Fig. 1a). CRISPR-Cas9-mediated gene knockout (KO) provided orthogonal evidence for *ADAR* dependency in these cell lines (Fig. 1b). In contrast, *ADAR* deletion did not induce significant cell lethality in *ADAR* KO-insensitive A549 cells (Fig. 1b and Supplementary Fig. 1a).

*ADAR* encodes multiple isoforms of the ADAR1 protein, including a constitutively expressed, predominantly nuclear p110 isoform and an interferon-inducible, nuclear and cytoplasmic p150 isoform^10,11^. ADAR1 edits adenosine (A) to inosine (I) in RNA. This editing function is thought to destabilize regions of RNA duplexes that are formed by inverted repetitive elements within RNAs^10–15^. In this manner, A-to-I editing has been proposed to prevent cytoplasmic RNA sensors of the innate immune system, such as MDA5 and PKR, from erroneously recognizing endogenous dsRNA as foreign^11–15^. Indeed, germline mutations in human *ADAR* cause Aicardi-Goutières syndrome, which is characterized by constitutive activation of type I interferon (IFN-I) signaling and widespread autoinflammation^16^, highlighting the role of ADAR1 in curbing aberrant IFN-I responses.

To investigate the importance of the IFN-I pathway in ADAR1*-*dependent lung cancer cell lines, we performed differential gene expression analysis on publicly available RNA sequencing (RNA-seq) data derived from cancer cell lines in the Cancer Cell Line Encyclopedia (CCLE)^17^. Consistent with the known role of ADAR1 in IFN-I signaling, we found higher expression of ISGs in ADAR1-dependent versus non-ADAR1-dependent lung cancer cell lines (Supplementary Data 1), suggesting that an elevated interferon gene expression signature may serve as a biomarker for ADAR1 dependency. Next, we identified additional lung cancer cell lines with elevated interferon gene expression signatures, none of which had been analyzed in published shRNA or CRISPR-Cas9 loss-of-function screens (Supplementary Data 2). Similar to the ADAR1-dependent cell lines identified in the genome-scale shRNA screening datasets, these cell lines (NCI-H596, HCC1438, and SW900) also expressed high levels of interferon-inducible proteins, such as PKR and MDA5 (Fig. 1c), secreted IFN-β spontaneously (Fig. 1d), and were sensitive to ADAR1 loss (Fig. 1e). Of note, this pattern of ADAR1 dependency was not restricted to lung cancer cell lines. Analysis of a CRISPR-Cas9 screen^18^ identified an ADAR1-dependent pancreatic cancer cell line, PATU-8902, which also expressed high levels of interferon-inducible proteins (Fig. 1c, d and Supplementary Fig. 1b).

Next, we sought to determine whether activation of IFN-I signaling in the context of ADAR1 deficiency could induce cell lethality in non-ADAR1-dependent cancer cell lines. We deleted *ADAR* in A549 and NCI-H1437 cells, which normally tolerate ADAR1 deficiency, and then treated these cell lines with IFN-I. Treatment with either interferon-α (IFN-α) or interferon-β (IFN-β) caused increased cell lethality in ADAR1-deficient A549 and NCI-H1437 cells as compared to vehicle-treated controls (Fig. 1f and Supplementary Fig. 1c). We observed similar results in an extended panel of *ADAR* KO-insensitive cancer cell lines treated with IFN-β, although the magnitude of the cell lethality phenotype varied between cell lines (Supplementary Fig. 1d). ADAR1-deficient A549 cells treated with IFN-β showed increased caspase 3/caspase 7 activity, indicative of increased apoptosis (Supplementary Fig. 1e). These data demonstrate that activation of IFN-I signaling in the setting of ADAR1 deficiency can induce cell lethality in normally *ADAR* KO-insensitive cancer cell lines.

To establish whether IFN-I signaling is required for ADAR1 dependency, we deleted a component of the IFN-I receptor, *IFNAR1*, in ADAR1-dependent cell lines prior to *ADAR* knockout. We confirmed that *IFNAR1* deletion decreased IFN-I signal transduction as shown by decreased STAT1 phosphorylation and decreased upregulation of the ISGs MDA5 and ISG15 in response to exogenous IFN-β stimulation (Supplementary Fig. 2a). *IFNAR1* knockout did not substantially rescue cell lethality induced by *ADAR* deletion in either HCC366 or NCI-H1650 cells (Fig. 1g and Supplementary Fig. 2b, c), suggesting that IFN-I signaling is not necessary for the cell lethality caused by *ADAR* deletion in these cell lines. Therefore, an elevated interferon gene expression signature appears to be a biomarker for vulnerability to ADAR1 loss.

### ADAR1 dependency does not require MDA5/MAVS signaling

**Fig. 2:**
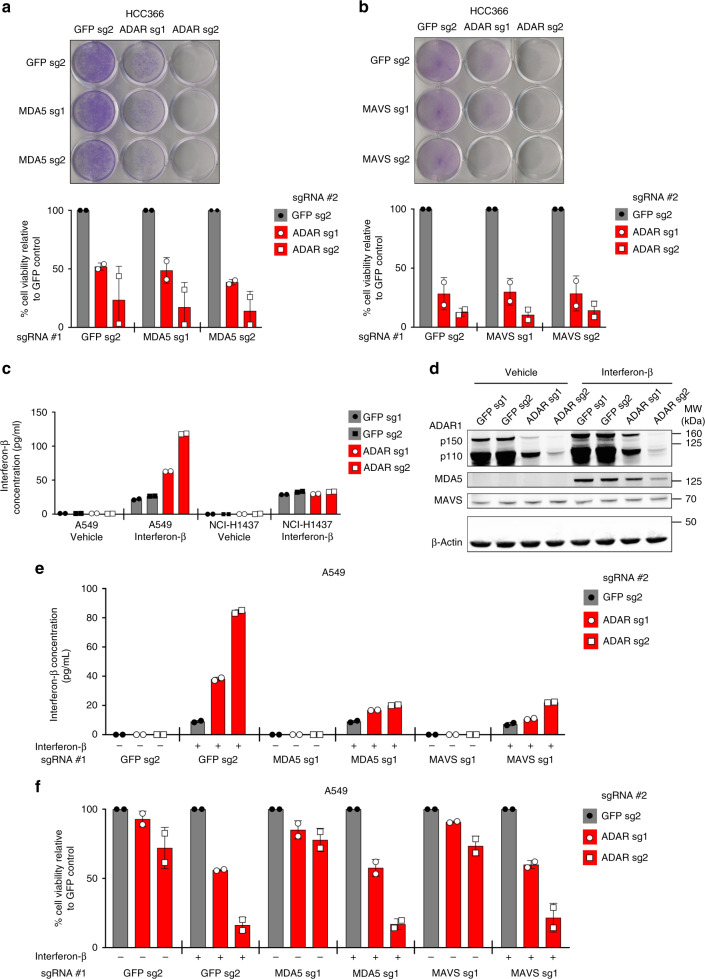
MDA5 and MAVS are required for IFN-induced IFN-β production, but not cell lethality, after *ADAR* deletion. **a**, **b** Cell viability of control and MDA5-deficient (**a**) or MAVS-deficient (**b**) HCC366 cells was assessed by crystal violet staining 8–13 days after *GFP* or *ADAR* KO with CRISPR-Cas9. A representative image of crystal violet staining (top) and quantitation of cell viability (bottom) from two independent biological replicates (for both **a** and **b**) are shown. Cell viability values were normalized to the GFP sg2 control #2 within each group of isogenic cell lines. **c** IFN-β secretion by control or ADAR1-deficient A549 cells was measured by ELISA after treatment with either vehicle or IFN-β (10 ng/mL) for 24 h. NCI-H1437 cells harbor a homozygous deletion of the *IFNB1* locus. Technical replicates from one representative experiment are shown. Three independent biological replicates were performed for A549 cells and one experiment was performed for NCI-H1437 cells. **d** Immunoblots showing MDA5 and MAVS protein levels in control (GFP sgRNAs) and ADAR1-deficient A549 cells 24 h after treatment with vehicle or IFN-β (10 ng/mL). β-Actin served as a loading control. One representative immunoblot from two independent biological replicates is shown. **e** IFN-β secretion by the indicated A549 cells was measured by ELISA after treatment with vehicle or IFN-β (10 ng/mL). Technical replicates from one representative experiment out of two independent biological replicates are shown. **f** Cell viability of the indicated A549 cells from **e** was assessed by cell counting 2 days after treatment with vehicle or IFN-β (10 ng/mL). Cell viability values were normalized to the GFP sg2 control #2 within each group of vehicle or IFN-β-treated isogenic cell lines. Two independent biological replicates are shown. Error bars represent standard deviation in all graphs

To determine the mechanism of cell lethality after *ADAR* deletion, we examined whether innate immune sensing pathways known to act downstream of ADAR1 were necessary for ADAR1 dependency. Prior studies have demonstrated that ADAR1 suppresses the activation of the cytoplasmic dsRNA sensor MDA5, which signals to MAVS to upregulate expression of ISGs in response to specific types of dsRNA^11–13,19^. Deletion of MDA5 or MAVS has also been shown to rescue embryonic lethality in *Adar1*^*−/−*^ mice^11,12,19^. Notably, MDA5 gene expression and protein levels correlate with *ADAR* KO-sensitivity across a spectrum of cancer cell lines (Fig. 1c and Supplementary Fig. 3a, b). However, deletion of neither MDA5 nor MAVS could rescue cell lethality after *ADAR* knockout in the cell lines tested (Fig. 2a, b and Supplementary Fig. 3c–f), indicating that MDA5/MAVS signaling is dispensable for this phenotype.

Consistent with the known role of ADAR1 in suppressing IFN-β expression^12,20^, ADAR1-deficient A549 cells secreted increased amounts of IFN-β in response to IFN-β stimulation (Fig. 2c), whereas control A549 cells did not. NCI-H1437 cells served as an additional control in this experiment since this cell line displays homozygous loss of the *IFNB1* gene. Notably, IFN-β treatment upregulated MDA5 protein levels in A549 cells (Fig. 2d), suggesting that MDA5 may mediate this phenomenon of interferon-induced interferon production. Deletion of either MDA5 or MAVS prior to *ADAR* knockout abrogated the interferon-induced interferon production phenotype observed in ADAR1-deficient A549 cells (Fig. 2e and Supplementary Fig. 3g) without affecting the cell lethality induced by the combination of ADAR1 depletion and IFN-β treatment (Fig. 2f and Supplementary Fig. 3h). Together, these data support the notion that *ADAR* knockout can trigger the activation of the IFN-I pathway (MDA5/MAVS-dependent) and the induction of cell lethality (MDA5/MAVS-independent) through distinct upstream mechanisms in cancer cell lines.

Since the MDA5/MAVS cytosolic RNA sensing pathway is not essential for *ADAR* genetic dependency, we next examined whether the cGAS/STING cytosolic DNA sensing pathway may mediate the cell lethality induced by ADAR1 loss. Indeed, we observed that *ADAR* KO-sensitive cell lines produced higher levels of the adaptor protein STING as compared to *ADAR* KO-insensitive cell lines (Supplementary Fig. 4a). Moreover, deletion of STING in *ADAR* KO-sensitive HCC366 cells resulted in decreased protein levels of ISGs such as MDA5 and ISG15 (Supplementary Fig. 4b). However, STING deletion did not rescue the cell lethality induced by ADAR1 inactivation in HCC366 cells (Supplementary Fig. 4c), suggesting that the cytosolic DNA sensing pathway is not essential for *ADAR* genetic dependency.

### PKR activation mediates cell lethality induced by ADAR1 loss

**Fig. 3:**
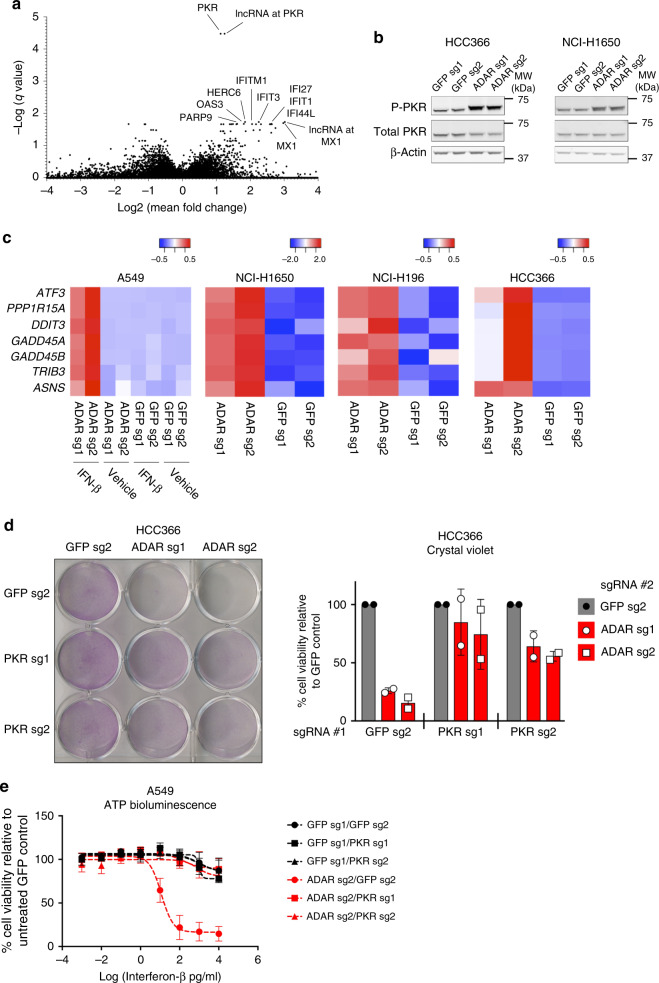
Cell lethality after *ADAR* deletion is partially mediated through activation of PKR signaling. **a** Analysis of differentially expressed genes between *ADAR* KO-sensitive and KO-insensitive cancer cell lines using gene expression data from CCLE^17^. Differentially expressed genes are plotted by −log(q-value) on the *y*-axis versus log2(fold change) on the *x*-axis. **b** Immunoblots showing phosphorylated (Thr-446) and total PKR protein levels 5 days after *ADAR* deletion by CRISPR-Cas9 for the indicated cell lines (*n* = 5). β-Actin served as a loading control. **c** Heat maps showing standardized *t*-statistics of normalized expression values for the indicated genes (rows) after deletion of *GFP* (control) or *ADAR* (columns) in the indicated cancer cell lines (*n* = 1). Color scales show relative normalized expression between *ADAR* and *GFP* KO samples. **d** Cell viability of control or PKR-deficient HCC366 cells was assessed by crystal violet staining 8 days after *GFP* or *ADAR* KO with CRISPR-Cas9. A representative image of crystal violet staining (left) and quantitation of cell viability (right) from two independent biological replicates are shown. Cell viability values were normalized to the GFP sg2 control #2 within each group of isogenic cell lines. **e** Cell viability of control, PKR-deficient, ADAR1-deficient, or ADAR1/PKR double-deficient A549 cells was assessed by ATP bioluminescence 3 days after treatment with vehicle or IFN-β (10 ng/mL). ATP bioluminescence values were normalized to the vehicle-treated control within each isogenic cell line. Data from two biological replicates are shown. Note**:** ADAR sg2 was used in this experiment. Error bars represent standard deviation in all graphs

As an alternative approach to interrogate the mechanisms underlying ADAR1 dependency, we performed differential gene expression analysis between an expanded set of ADAR1-dependent and non-ADAR1-dependent cancer cell lines of diverse lineages (Supplementary Data 3)^9,18,21^. The most statistically significant differentially expressed gene in ADAR1-dependent cell lines was *EIF2AK2*, which encodes the dsRNA-activated protein kinase, PKR (Fig. 3a). PKR is an antiviral cytoplasmic dsRNA sensor with kinase activity. Upon binding of dsRNA, PKR undergoes dimerization and auto-phosphorylation at threonine residue 446, which results in its activation^14,22^. Activated PKR initiates downstream signals to inhibit protein translation and induce apoptosis^14,23^.

Given the high expression of PKR in ADAR1-dependent cell lines, we hypothesized that PKR may mediate cell lethality after *ADAR* deletion. Indeed, we detected increased PKR auto-phosphorylation at threonine 446 after *ADAR* knockout in ADAR1-dependent cell lines (Fig. 3b and Supplementary Fig. 5a). In *ADAR* KO*-*insensitive cell lines (A549 and NCI-H1437), we observed increased levels of PKR phosphorylation only after the combination of ADAR1 depletion and IFN-β treatment (Supplementary Fig. 5b). Activated PKR is known to phosphorylate the eukaryotic translation initiation factor, eIF2α, and lead to activation of the ATF4 transcription factor^24^. RNA-seq analysis confirmed that expression of canonical ATF4-regulated genes, such as *PPP1R15A*, *ATF3*, *DDIT3*, *GADD45A*, *GADD45B*, *TRIB3*, and *ASNS*, were enriched after *ADAR* knockout in ADAR1-dependent cell lines (Fig. 3c). Moreover, the combination of *ADAR* deletion and IFN-β treatment induced a similar enrichment in the expression of ATF4-regulated genes in the non-ADAR1-dependent cell line, A549 (Fig. 3c). Together, these data show that signaling through PKR is activated after *ADAR* knockout.

To establish a mechanistic link between PKR and ADAR1 dependency, we deleted PKR in *ADAR* KO-sensitive HCC366 and NCI-H1650 cells prior to *ADAR* deletion (Supplementary Fig. 5c). Consistent with its known role in promoting apoptosis^14^, deletion of PKR partially rescued cell lethality induced by *ADAR* knockout (Fig. 3d and Supplementary Fig. 5d), although the magnitude of this rescue varied between the cell lines tested. Likewise, in normally *ADAR* KO-insensitive A549 cells, deletion of PKR partially rescued the cell lethality triggered by IFN-β treatment in the context of ADAR1 deficiency (Fig. 3e and Supplementary Fig. 5e). These data demonstrate that the cell lethality induced by *ADAR* knockout is mediated at least in part through activation of PKR signaling.

### Catalytically inactive ADAR1-p150 can prevent cell lethality

**Fig. 4:**
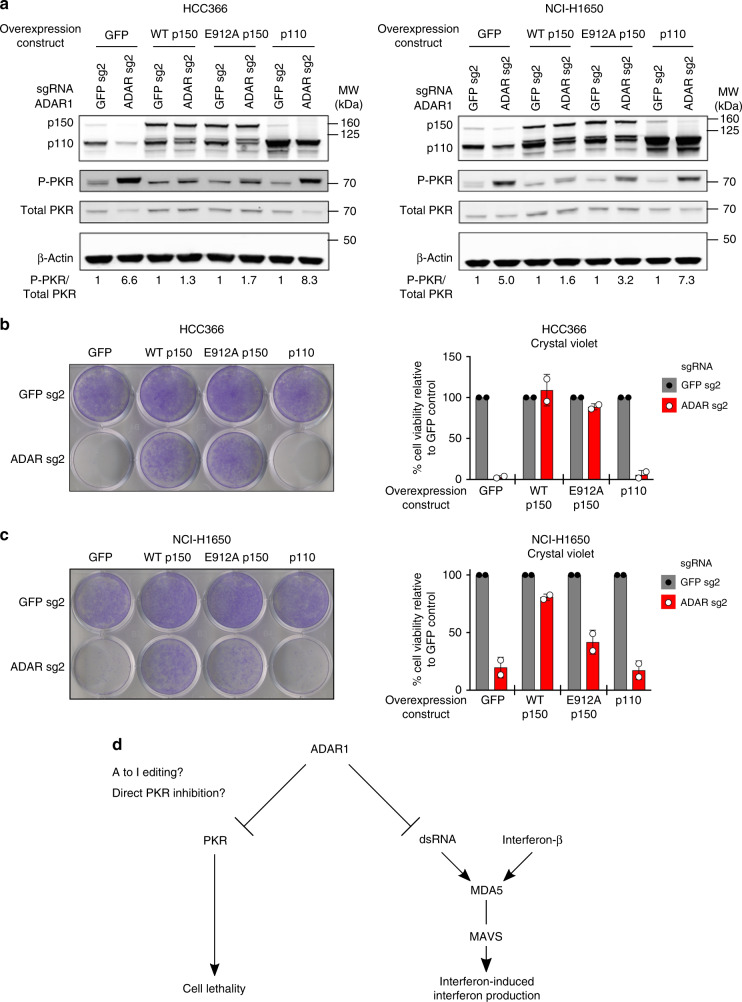
Both non-enzymatic and catalytic functions of ADAR1-p150 may be important to prevent cell lethality in cancer cell lines. **a** GFP control, WT ADAR1-p150, E912A ADAR1-p150, or WT ADAR1-p110 proteins were overexpressed in HCC366 (right) or NCI-H1650 cells (left) prior to transduction with lentivirus that co-expressed Cas9 and sgRNAs targeting *GFP* or *ADAR*. Protein lysates were collected 6 days after *GFP* or *ADAR* KO and were probed with antibodies against ADAR1, phospho-PKR, total PKR, and β-Actin (loading control) in HCC366 (left) or NCI-H1650 cells (right). The fold change in the phospho-PKR to total PKR ratio (P-PKR/Total PKR) relative to the corresponding GFP sgRNA control is shown in each lane (*n* = 2). **b** Cell viability of GFP control or ADAR1-overexpressing HCC366 cells was assessed by crystal violet staining 11–13 days after *GFP* or *ADAR* KO (using ADAR sg2) with CRISPR-Cas9. A representative image of crystal violet staining (left) and quantitation of cell viability (right) from two independent biological replicates are shown. Cell viability values were normalized to the GFP sg2 control within each pair of isogenic cell lines. **c** Cell viability of GFP-overexpressing (control) or ADAR1-overexpressing NCI-H1650 cells was assessed by crystal violet staining 13–16 days after *GFP* or *ADAR* KO (using ADAR sg2) with CRISPR-Cas9. A representative image of crystal violet staining (left) and quantitation of cell viability (right) from two independent biological replicates are shown. Cell viability values were normalized to the GFP sg2 control within each pair of isogenic cell lines. **d** Model of the pathways that mediate cell lethality and interferon-induced interferon production after *ADAR* deletion in cancer cell lines. Error bars represent standard deviation in all graphs

ADAR1 could inhibit PKR activation and prevent cell lethality in cancer cell lines through several distinct mechanisms^25,26^. Since dsRNA is a known ligand for PKR, one possibility is that ADAR1 prevents PKR activation by limiting the pool of cytoplasmic dsRNA through its catalytic deaminase function^27,28^. Alternatively, ADAR1 has been shown to interact directly with PKR and inhibit PKR activation after viral infection^25,26^. To differentiate between these possibilities, we first engineered different variants of ADAR1 to be resistant to CRISPR-Cas9-mediated silencing and overexpressed these variants in ADAR1-dependent cell lines. While overexpression of the wild-type (WT) p150 isoform of ADAR1 prior to deletion of endogenous ADAR1 prevented PKR phosphorylation and rescued cell lethality, overexpression of the WT p110 isoform had minimal effects on these phenotypes (Fig. 4a–c and Supplementary Fig. 6b–f). These data indicate that the p150 isoform of ADAR1 is critical for inhibiting PKR activation and preventing cell lethality in ADAR1-dependent cancer cell lines.

To examine whether the catalytic function of ADAR1 is necessary to prevent PKR activation and subsequent cell lethality, we generated a previously described catalytically inactive mutant of ADAR1-p150 (E912A)^16,29^ (Supplementary Fig. 6a). Overexpression of E912A ADAR1-p150 inhibited PKR phosphorylation and rescued cell lethality after *ADAR* deletion to a similar degree as WT ADAR1-p150 in HCC366 cells (Fig. 4a, b and Supplementary Fig. 6b, c, e). We observed similar results in NCI-H1650 cells, although the degree of rescue observed with E912A ADAR1-p150 in this cell line was less robust compared to WT ADAR1-p150 (Fig. 4a, c and Supplementary Fig. 6b, d, and f). Together, these data suggest that both catalytic and non-enzymatic functions of ADAR1 may be important to prevent cell lethality in a subset of cancer cell lines, although the relative contribution of each of these functions may be cancer cell line-specific.

## Discussion

In summary, we have identified *ADAR* loss as a genetic dependency in a subset of lung cancer cell lines which possess a high interferon gene expression signature. Although an elevated interferon gene expression signature is predictive of ADAR1-dependence, the mechanisms underlying this sensitivity to *ADAR* knockout will require further investigation. The p150 isoform of ADAR1 appears to prevent lethality in cancer cell lines at least partly through inhibition of the cytoplasmic RNA sensor PKR (Fig. 4d). Overexpression studies suggest that both the catalytic and non-enzymatic functions of ADAR1-p150 may contribute to limiting PKR activation and preventing cell lethality in ADAR1-dependent cancer cell lines. Distinct from the cell lethality phenotype, loss of ADAR1 primes cancer cell lines to produce IFN-β in response to IFN-β stimulation in a MDA5/MAVS-dependent manner (Fig. 4d).

Our data show that MDA5/MAVS signaling is not essential for *ADAR* genetic dependency in lung cancer cell lines. These data are distinct from previously published studies, which demonstrated a critical role for the MDA5/MAVS pathway in the embryonic lethality phenotype observed in *Adar1*^−/−^ mice^12,13,19^. Notably, this embryonic lethality results from failure of early erythropoiesis. One explanation for this difference is that the downstream pathways that mediate cellular lethality after *ADAR* deletion may vary depending on the specific cell type, developmental stage, and/or malignant nature of the cells under investigation.

Furthermore, a prior study reported that RNaseL can mediate cellular lethality after *ADAR* deletion in A549 cells^30^. Using these published sgRNAs to delete *ADAR* in A549 cells (Supplementary Fig. 7), we did not observe any significant changes in cellular viability, as evidenced by the fact that we could harvest substantial amounts of protein from ADAR1-deficient A549 cells after *ADAR* knockout. One possible interpretation is that genetic drift of A549 cells passaged in different laboratories may explain these discrepant results^31^.

ADAR1 represents a unique therapeutic target as loss of its activity can cause both cell-intrinsic lethality and the induction of a key anti-tumor cytokine. Inhibition of ADAR1 could directly kill a subset of cancers that express high levels of ISGs via the activity of PKR. Furthermore, ADAR1 inhibition might amplify the IFN-I response in the tumor microenvironment by triggering IFN-β production by tumor cells. Thus, ADAR1 inhibitors could synergize with existing cancer immunotherapies through stimulation of cytotoxic T and natural killer cells (J. Ishizuka, R.T. Manguso, and W.N. Haining, personal communication).

Our study suggests several possible approaches to disrupt the function of ADAR1 in cancer cells: direct enzymatic inhibition of its adenosine deaminase activity and/or inactivation of non-enzymatic functions unique to the p150 isoform, such as direct PKR binding. Further experiments will be required to determine the utility of each of these approaches for cancer therapeutics.

## Methods

### Cell lines and culture conditions

Cancer cell lines were grown and maintained in RPMI media supplemented with 10% fetal bovine serum (FBS), and 1% penicillin, streptomycin, and l-glutamine. The following cancer cell lines were obtained from the CCLE: A549, NCI-H460, NCI-H1299, NCI-H1437, RERFLCAI, HCC366, HCC1438, NCI-H196, NCI-H596, NCI-H1650, and SW900 (lung); PATU-8902 (pancreas); RKO (colorectal); AGS (stomach); BT20 (breast); and RKN (soft tissue). Prior to shipping each cell line, the CCLE performed cell line authentication with DNA fingerprinting and mycoplasma testing.

### CRISPR-Cas9 gene knockout

Single guide RNA sequences were designed using the sgRNA Designer tool on The RNAi Consortium (TRC) portal (http://portals.broadinstitute.org/gpp/public/analysis-tools/sgrna-design). sgRNA sequences are displayed in Supplementary Table 2. sgRNAs were cloned into the Cas9-expressing lentiviral vector CRISPRv2 (http://www.genome-engineering.org/crispr). For lentivirus production, individual CRISPRv2 vectors were introduced along with packaging vectors into 293 T cells via calcium phosphate transfection according to the manufacturer’s instructions (Clontech). Lentivirus was harvested at 48 and 72 h after transfection in RPMI media supplemented with 10% FBS and filtered with 45 μm filters before transduction of cancer cell lines. Transduced cell lines were selected in 2 μg/mL puromycin and/or 10 μg/mL blasticidin for at least 5 days prior to use in assays. Thereafter, protein lysates were collected from the transduced cells and protein levels of the targeted gene(s) were assessed by immunoblotting. For cell lines in which two genes were knocked out, stable single KO cell lines maintained under 2 μg/mL puromycin selection were transduced with lentivirus expressing Cas9, a second guide RNA, and a blasticidin resistance marker. Double KO cells were selected with both 2 μg/ml puromycin and 10 μg/ml blasticidin for at least 5 days prior to use in assays.

### Cell viability assays

Cell counting was performed using a Vi-Cell XR Cell Counter (Beckman-Coulter). For ATP bioluminescence experiments, cells were plated at a density of 3000 cells per well in 96-well assay plates (Corning). ATP bioluminescence was assessed at 3 and 6 days after plating with the CellTiter-Glo Luminescent Cell Viability Assay (Promega). For crystal violet staining, cells were plated at a density ranging from 25,000 to 100,000 cells per well in 12-well tissue culture plates. Once the GFP control cells grew to near confluency, each well was washed twice with ice cold PBS, fixed with ice cold methanol for 10 min on ice, stained with 0.5% crystal violet solution (made in 25% methanol) for 10 min at room temperature, and washed at least four times with water. To quantify cell viability in crystal violet stained plates, images were obtained from five fields of each well at ×40 magnification with an Olympus inverted microscope. The number of cell nuclei in each image was counted using ImageJ and averaged for each well. All cell viability assays were performed in triplicate.

### Antibodies and immunoblotting

Cells were lysed in RIPA lysis buffer (Thermo Fisher Scientific) supplemented with 1× protease and phosphatase inhibitor cocktails (Roche). Protein concentrations were obtained using the BCA Protein Assay Kit (Pierce) and normalized between all samples. Protein extracts were analyzed by standard immunoblotting with the following primary antibodies: ADAR1 (#14175), ISG15 (#2758), MAVS (#3993), MDA5 (#5321), STING (#13647), total PKR (#3072), and phospho-STAT1 Tyr701 (#9167) from Cell Signaling; phospho-PKR Thr-446 (ab32036) from Abcam; IFNAR1 (A304-290A) and total STAT1 (A302-752A) from Bethyl; and β-actin (sc-47778) from Santa Cruz. All antibodies were used at a dilution of 1:1000 except β-actin which was used at a dilution of 1:4000 or 1:5000. The following secondary antibodies were used at a 1:5000 or 1:10,000 dilution: Goat anti-Rabbit IRDye 800CW (LI-COR, 926-32211) and Goat anti-Mouse IRDye 680LT (LI-COR, 926-68020) from LI-COR Biosciences. Immunoblots for phospho-PKR and phospho-STAT1 were stripped with Restore Western Blot Stripping buffer (Thermo Fisher Scientific, #21059) prior to immunoblotting with total PKR and total STAT1, respectively. Immunoblots were imaged using the LI-COR digital imaging system and ImageJ. Quantitation of band intensities was performed with ImageJ. All immunoblots were cropped to optimize clarity and presentation. Uncropped scans of the immunoblots presented in the main figures of this manuscript are shown in Supplementary Fig. 8.

### Enzyme-linked immunosorbent assay

IFN-β detection was performed with the VeriKine-HS Human IFN Beta Serum ELISA Kit (PBL Assay Science) according to the manufacturer’s instructions. To detect spontaneous IFN-β production, 4 × 10^5^ cells were seeded in six-well culture plates on day 1. On day 2, the culture media was replaced with 1.5 mL of fresh media for each well. On day 3, 200 μL of conditioned media from each well was collected and spun to pellet cells. Fifty microliters of conditioned media was assayed in duplicate for each sample. Fresh RPMI media was used as the diluent for all standards and blanks. All concentrations of IFN-β were calculated according to the standard curve generated in each experiment. For exogenous IFN-I treatment experiments, 2 × 10^5^ cells were seeded in six-well culture plates on day 1. On day 2, the culture media was replaced with media supplemented with recombinant IFN-α or IFN-β (both at 10 ng/ml). On day 3, the culture media was replaced with 1.5 mL fresh media for each well. On day 4, the enzyme-linked immunosorbent assay was performed on the conditioned media as described above.

### Interferon treatment

For interferon treatment assays, cells were plated at a density of 3000 cells per well in a 96-well assay plate (Corning). The following day, cells were treated with human interferon-alpha 1 (Cell Signaling, #8927) or recombinant human IFN-beta 1a (mammalian) protein (PBL Assay Science, #114151). Control wells were treated with sterile water (vehicle). Cell viability was assayed 3 days after IFN-I treatment with CellTiter-Glo Luminescent Cell Viability Assay (Promega). The ATP bioluminescence values of the treated wells were normalized to those of the vehicle-treated controls. Apoptosis was assayed 3 days after IFN-I treatment with the Caspase-Glo 3/7 Assay (Promega). Dose curves were obtained using least-squares nonlinear regression on a standard four-parameter logistic model using GraphPad Prism 7 software.

### RNA-sequencing and analysis

For GFP control or ADAR1-deficient A549 cell lines, cells were treated with 10 ng/mL IFN-β or sterile water (vehicle) for 24 h before RNA isolation. For HCC366, NCI-H1650, and NCI-H196 cells, RNA was isolated 5 days after transduction with lentivirus co-expressing Cas9 and sgRNAs targeting *GFP* or *ADAR*. RNA was isolated using the RNeasy Kit (Qiagen) with on-column DNase I treatment followed by ribosomal RNA depletion using the NEBNext rRNA Depletion Kit (E6310). RNA sequencing libraries were prepared using the NEBNext Ultra Directional RNA Library Prep Kit (E7420S) and sequenced on the Illumina HiSeq instrument (150-bp paired-end reads). Alignment against the human genome (hg19) was performed using the STAR aligner^32^. Reads were quantified using HTSeq^33^ and each gene was then fit with a generalized linear model using DESeq2 (ref. ^34^).

Heat maps showing standardized *t*-statistics (*T*_*ij*_) of normalized expression values for each sample per gene were calculated using *T*_*ij*_ = (*x*_*ij*_–*x*_*j*_)/(*s*_*j*_/√*n*), where *x*_*ij*_ = normalized expression value for sample *i* and gene *j*, *x*_*j*_ = sample mean for normalized expression of gene *j*, *s*_*j*_ = sample standard deviation for normalized expression of gene *j*, *n* = number of samples.

To generate interferon gene expression signature scores for cancer cell lines in the CCLE (Supplementary Data 2), a list of 27 ISGs (columns in Supplementary Data 2) that were significantly differentially expressed between *ADAR* KO-sensitive and KO-insensitive lung cancer cell lines was compiled from Supplementary Data 1. Interferon gene expression signature scores were computed by taking the sum of log2(*x* + 1) transformed RPKM expression values across all 27 genes in the signature. These raw sums were standardized and the *z*-scores were reported as the final interferon gene expression signature scores for each of the CCLE cell lines (Supplementary Data 2).

### ADAR1 mutagenesis and overexpression

An *ADAR* open-reading frame (ORF) clone was obtained from GeneCopoeia (C0744). The entire ORF was sequenced to confirm fidelity to the NCBI Reference Sequence NM_001111.4. Entry clones for both ADAR1 p110 and p150 were obtained through PCR-amplification of the *ADAR* ORF and were sub-cloned into a Gateway donor vector. Using the QuikChange Lightning Site-Directed Mutagenesis Kit (Agilent), silent mutations were introduced separately into the protospacer adjacent motif (PAM) sequences targeted by *ADAR* sgRNA 1 and sgRNA 2 to render the constructs resistant to CRISPR-Cas9 editing by these sgRNAs. Subsequently, the E912A mutation was engineered into each CRISPR-Cas9-resistant ADAR1 construct separately using site-directed mutagenesis. The resulting CRISPR-Cas9-resistant ADAR1 constructs and a GFP control construct were sub-cloned into the pLX307 lentiviral expression vector (Addgene) under the control of an EF-1α promoter. Each expression vector was then transfected into 293 T cells to generate lentivirus. Lentiviral transduction of target cell lines was performed as described above.

### Statistical analysis

No statistical methods were used to predetermine sample size. Investigators were not blinded to sample allocation for any of the experiments. Data in all graphs are presented as the mean of either independent biological or technical replicates, as indicated in the figure legends, with all error bars representing standard deviation. The Krusal–Wallis test was utilized in Fig. 1b. The two-way ANOVA with Dunnett’s multiple comparisons post-test was utilized for Fig. 1f and Supplementary Fig. 1c. Pearson’s correlation coefficient (*R*^2^) was calculated by linear regression analysis in Supplementary Fig. 3b. Statistical comparisons of differential gene expression between groups of *ADAR* KO-sensitive and KO-insensitive cell lines were made with Mann–Whitney *U* tests in Supplementary Data 1 and 3. *p*-values less than 0.05 were considered to be statistically significant. All statistical tests were performed using R version 3.4.1 or GraphPad Prism 7 software.

## Supplementary information

Supplementary Data 1

Supplementary Data 2

Supplementary Data 3

Reporting Summary

Supplementary Information

Description of Additional Supplementary Files

## Data Availability

The raw and processed RNA-seq data presented in Fig. 3c can be accessed at the NCBI Gene Expression Omnibus repository (accession code GSE122168). The authors declare that all other data supporting the findings of these study are presented in Supplementary Table 1 and Supplementary Data 1–3. The source data used to generate Fig. 1a were obtained from publicly available shRNA screening datasets (Achilles v2.20.2 ExpandedGeneZSolsCleaned.csv) located in the Project Achilles Data Portal [http://portals.broadinstitute.org/achilles/]^9,18^. Additional publicly available *ADAR* knockdown data were obtained from the Project DRIVE Data Portal as of 31 August 2017 [https://oncologynibr.shinyapps.io/drive/]^21^. Publicly available CRISPR-Cas9 screening datasets (Achilles v3.3.8 Achilles_v3.3.8.Gs.gct) were also obtained from the Project Achilles Data Portal [http://portals.broadinstitute.org/achilles/]^9,18^. RNA-seq gene expression data (CCLE_RNAseq_081117.rpkm.gct) used to generate Fig. 3a, Supplementary Fig. 3b, and Supplementary Data 1 through 3 were obtained from the publicly available CCLE Data Portal [http://www.broadinstitute.org/ccle]^17^. A reporting summary for this Article is available as a Supplementary Information file.
