# Author Correction: Identification of ADAR1 adenosine deaminase dependency in a subset of cancer cells

**DOI:** 10.1038/s41467-022-29467-2

**Published:** 2022-04-01

**Authors:** Hugh S. Gannon, Tao Zou, Michael K. Kiessling, Galen F. Gao, Diana Cai, Peter S. Choi, Alexandru P. Ivan, Ilana Buchumenski, Ashton C. Berger, Jonathan T. Goldstein, Andrew D. Cherniack, Francisca Vazquez, Aviad Tsherniak, Erez Y. Levanon, William C. Hahn, Matthew Meyerson

**Affiliations:** 1 Department of Medical Oncology, Dana-Farber Cancer Institute, Boston, MA 02215 USA; 2 Broad Institute of Harvard and MIT, Cambridge, MA 02142 USA; 3 Department of Gastroenterology and Hepatology, University of Zurich and University Hospital Zürich, Zürich, 8091 Switzerland; 4 Department of Medical Oncology and Hematology, University Hospital Zurich, University of Zurich, 8091 Zurich, Switzerland; 5 The Mina and Everard Goodman Faculty of Life Sciences, Bar-Ilan University, 52900 Ramat Gan, Israel; 6 Department of Medicine, Brigham and Women’s Hospital, Harvard Medical School, Boston, MA 02115 USA; 7 Center for Cancer Genome Discovery, Dana-Farber Cancer Institute, Boston, MA 02215 USA; 8 Department of Pathology, Harvard Medical School, Boston, MA 02115 USA

Correction to: *Nature Communications* 10.1038/s41467-018-07824-4, published online 21 December 2018.

The original version of the Supplementary Information associated with this Article contained an error in Supplementary Fig. 4a. In Supplementary Fig. 4a the labels “ADAR KO sensitive” and “ADAR KO insensitive” at the top of the immunoblots were mistakenly swapped. The HTML version of the Article has been updated to include a corrected version of the Supplementary Information.

## Supplementary information

Updated Supplementary Information

