# Supplementary material for: Identification of ADAR1 adenosine deaminase dependency in a subset of cancer cells
Source: Nat Commun. 2018 Dec 21;9:5450. doi: 10.1038/s41467-018-07824-4 (PMC6303303; doi:10.1038/s41467-018-07824-4)
Supplement: Supplementary file 5 — Supplementary Information [file 41467_2018_7824_MOESM5_ESM.pdf]

Identification of ADAR1 adenosine deaminase dependency in a subset of cancer cells

Gannon et al.

Supplementary Information

The supplementary information contains 8 Supplementary Figures, 2 Supplementary Tables, and Supplementary References.

Supplementary Figures

Supplementary Figure 1

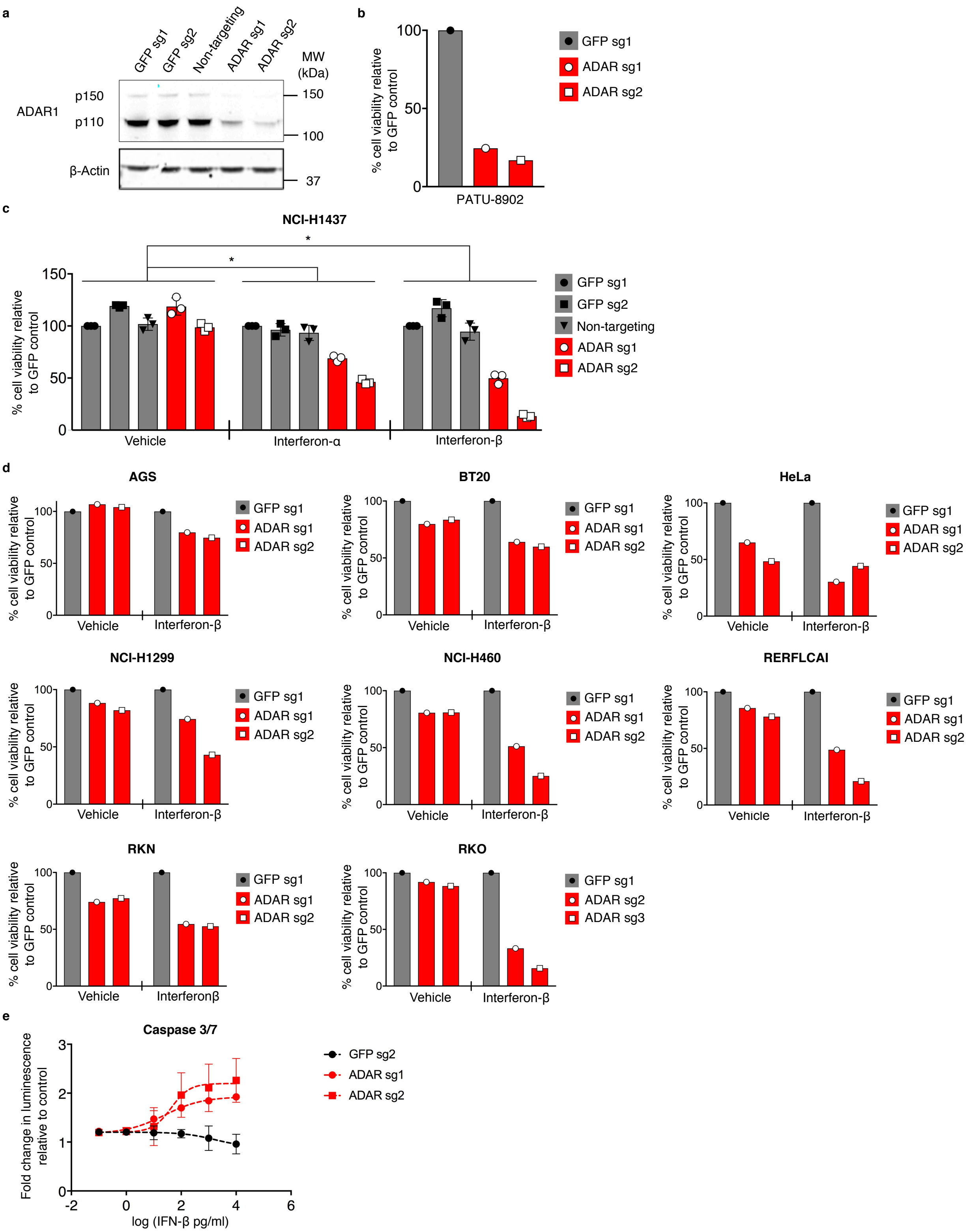

Supplementary Figure 1. Activation of IFN-I signaling in the setting of *ADAR* deletion can induce cell lethality in *ADAR* KO-insensitive cancer cell lines. This figure is related to Figure 1. (**a**) Immunoblot showing ADAR p150 and p110 protein levels in A549 cells 10 days after transduction with lentivirus expressing both Cas9 and sgRNAs targeting either control genes or *ADAR* (n=3). β-Actin served as a loading control. (**b)** Cell viability of PATU-8902 cells was assessed by ATP bioluminescence 11 days after *GFP* or *ADAR* KO with CRISPR-Cas9. ATP bioluminescence values were normalized to the GFP sg1 control (n=1). (**c**) Cell viability of control or ADAR1-deficient NCI-H1437 cells was assessed by ATP bioluminescence 3 days after treatment with vehicle or IFN-I (10 ng/mL). ATP bioluminescence values were normalized to the GFP sg1 control within each treatment group. Three independent biological replicates were performed. Two-way ANOVA showed a significant interaction between *ADAR* KO and IFN-I treatment (*p<0.0001). Dunnett's multiple comparisons post-test showed a significant difference between vehicle and IFN-I treatment groups and between control and *ADAR* KO groups (*p<0.0001, degrees of freedom=8, F-ratio=10.51). (**d**) Cell viability of control or ADAR1-deficient cancer cell lines was assessed by ATP bioluminescence 3 days after treatment with vehicle or IFN-β (10 ng/mL) (n=1 for each cell line). ATP bioluminescence values were normalized to the GFP sg1 control within each group of treated cells. (**e**) Caspase 3/7 activity of control or ADAR1-deficient A549 cells was assessed by caspase luminescence 3 days after treatment with vehicle or IFN-β (10 ng/mL). The values on the y-axis represent fold change in luminescence compared to the vehicle-treated control within each isogenic cell line. Data from two independent biological replicates are shown. Error bars represent standard deviation in all graphs.

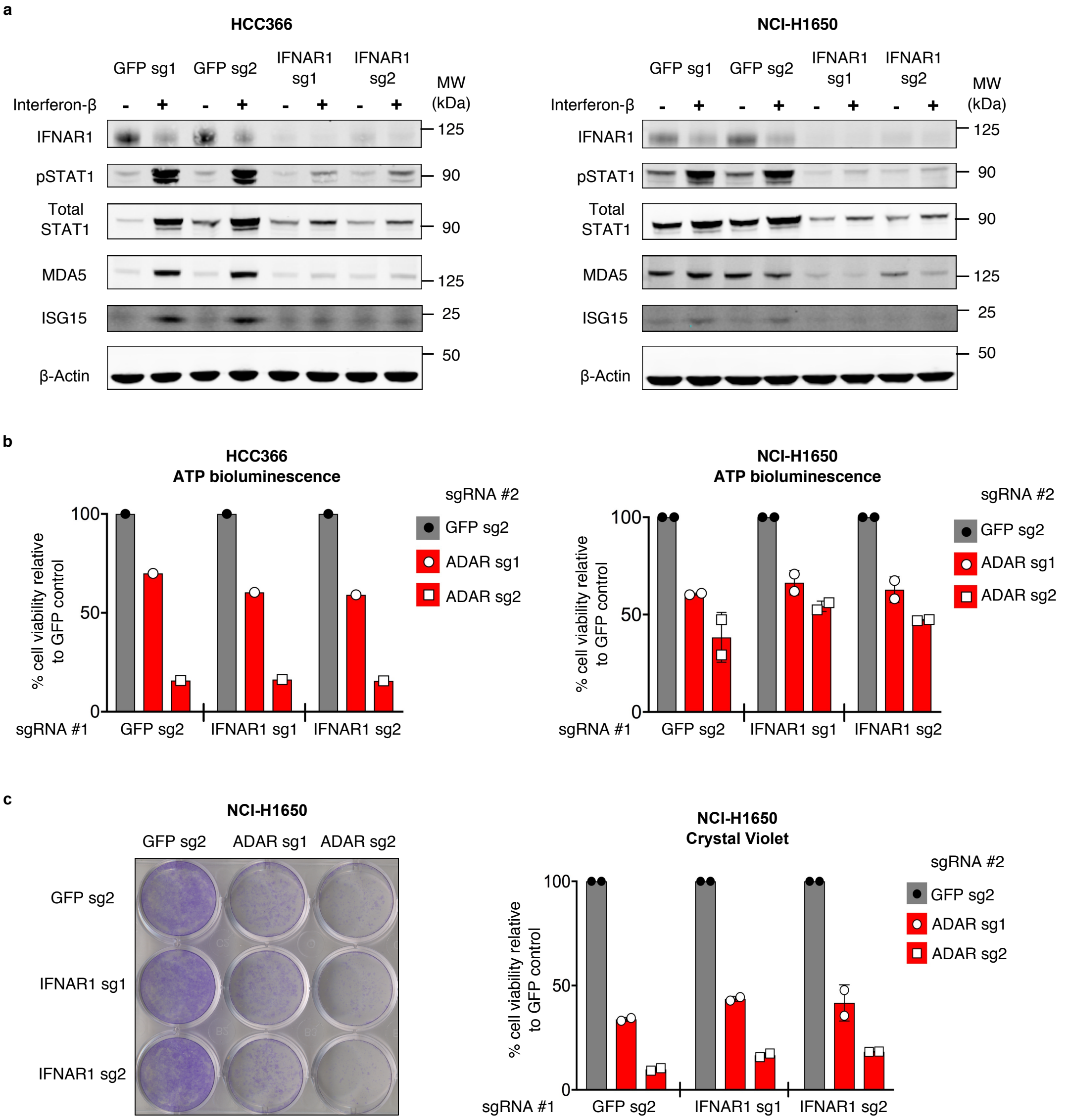

Supplementary Figure 2. IFN-I signaling is not necessary for cell lethality induced by *ADAR* deletion in ADAR1-dependent cancer cell lines. This figure is related to Figure 2. (**a**) Immunoblots showing IFNAR1, phospho-STAT1, total STAT1, MDA5, and ISG15 protein levels in control or IFNAR1-deficient HCC366 (left) and NCI-H1650 (right) cells 24 hours after treatment with vehicle or IFN-β (10 ng/mL) (n=2 for HCC366 and n=1 for NCI-H1650). β-Actin served as a loading control. (**b**) Cell viability of control or IFNAR1-deficient HCC366 (left) and NCI-H1650 (right) cells was assessed by ATP bioluminescence 9 days after *GFP* or *ADAR* KO with CRISPR-Cas9 (n=1 for HCC366, n=2 for NCI-H1650). ATP bioluminescence values were normalized to the GFP sg2 control #2 within each group of isogenic cell lines. (**c**) Cell viability of control or IFNAR1-deficient NCI-H1650 cells was assessed by crystal violet staining 13 to 15 days after *GFP* or *ADAR* KO with CRISPR-Cas9. A representative image of crystal violet staining (left) and quantitation of cell viability (right) from two independent biological replicates are shown. Cell viability values were normalized to the GFP sg2 control #2 within each group of isogenic cell lines. Error bars represent standard deviation in all graphs.

Supplementary Figure 3

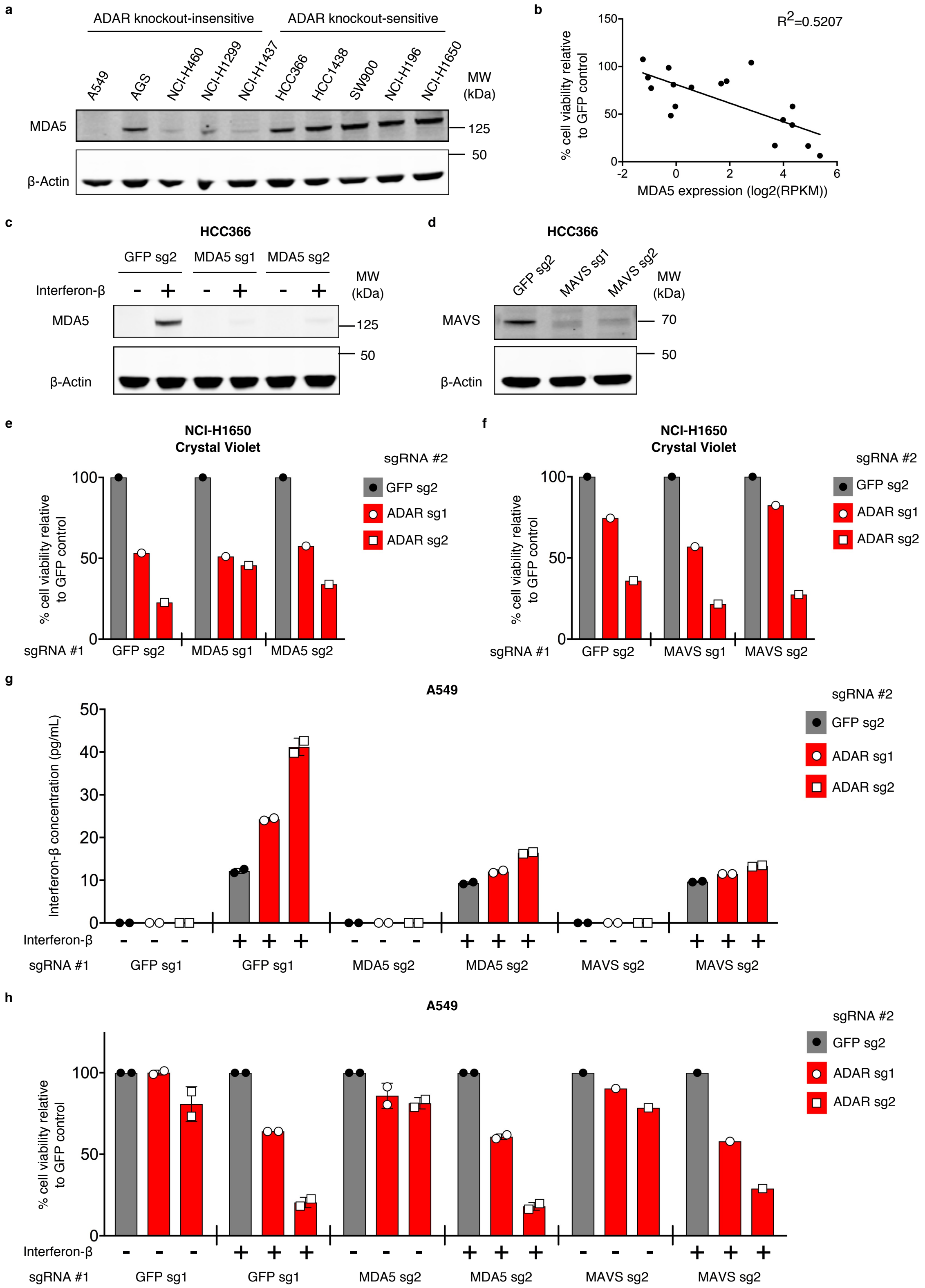

Supplementary Figure 3. MDA5 and MAVS are required for IFN-induced IFN-β production, but not cell lethality, after *ADAR* deletion. This figure is related to Figure 2. In this figure, panels (**g**) and (**h**) present similar data as panels (**e**) and (**f**) in Figure 2, but utilize different sgRNAs targeting MDA5 (sg2) and MAVS (sg2). (**a**) Immunoblot showing MDA5 protein levels in *ADAR* KO-insensitive and KO-sensitive cell lines (n=2). β-Actin served as a loading control. (**b**) MDA5 mRNA expression (x-axis), measured by reads per kilobase million (RPKM) and displayed as log2(RPKM), is plotted against the cell viability (relative to GFP sgRNA controls) of a panel of *ADAR* KO-insensitive (A549, AGS, BT20, NCI-H460, NCI-H1299, NCI-H1437, RERFLCAI, HeLa, RKN, and RKO) and KO-sensitive (HCC366, HCC1438, NCI-H196, NCI-H596, NCI-H1650, PATU-8902, and SW900) cancer cell lines as assessed by ATP bioluminescence 9 days after CRISPR-Cas9-mediated *GFP* or *ADAR* KO using ADAR sg2 (y-axis). Pearson correlation coefficient (R^2) is 0.5207 as calculated by linear regression analysis. (**c**, **d**) Validation of MDA5 (**c**) and MAVS (**d**) deletion by immunoblotting in HCC366 cells after CRISPR-Cas9-mediated gene KO. In (**c**), protein lysates were harvested 24 hours after vehicle or IFN-β treatment (10 ng/mL). β-Actin served as a loading control. (**e, f**) Cell viability of control and MDA5-deficient (**e**) or MAVS-deficient (**f**) NCI-H1650 cells was assessed by crystal violet staining 8 days after *GFP* or *ADAR* KO with CRISPR-Cas9. Quantitation of cell viability is shown (n=1 for both **e** and **f**). Cell viability values were normalized to the GFP sg2 control #2 within each group of isogenic cell lines. (**g**) IFN-β secretion by the indicated A549 cells was measured by ELISA after treatment with vehicle or IFN-β (10 ng/mL). Technical replicates from one representative experiment are shown (n=2 for MDA5 sg2, n=1 for MAVS sg2 for both (**g**) and (**h**)). (**h**) Cell viability of the indicated A549 cells from (**g**) was assessed by cell counting 2 days after treatment with vehicle or IFN-β (10 ng/mL). Cell viability values were normalized to the GFP sg2 control #2 within each group of isogenic cells. Error bars represent standard deviation in all graphs.

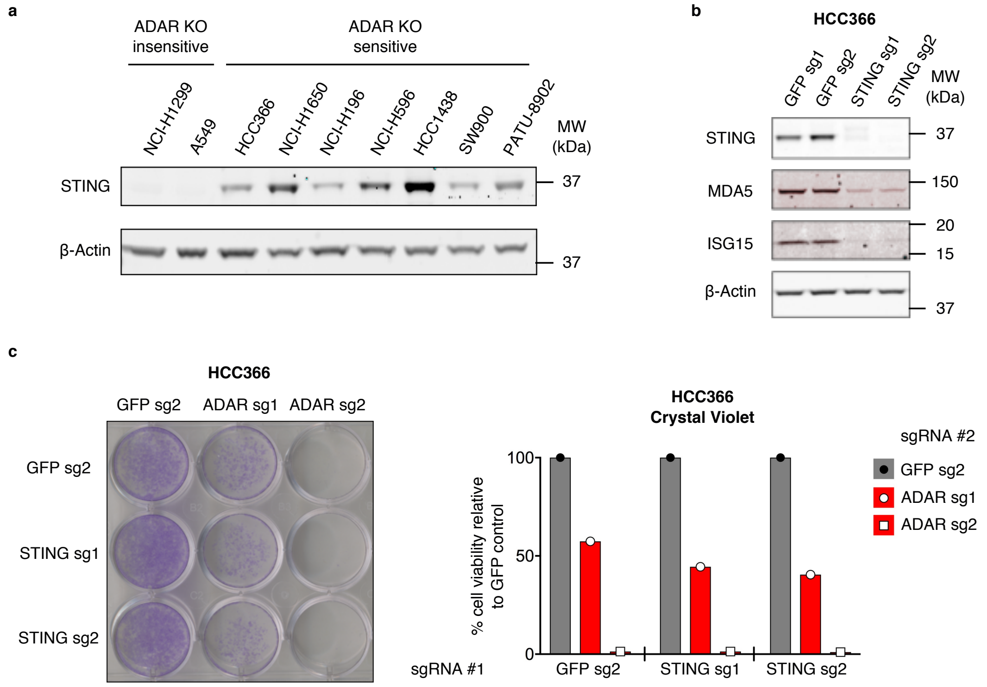

Supplementary Figure 4. STING deficiency does not prevent cell lethality after *ADAR* deletion in HCC366 cells. (**a**) Immunoblot showing STING protein levels in *ADAR* KO-insensitive and KO-sensitive cancer cell lines (n=2). β-Actin served as a loading control. (**b**) Immunoblots showing STING, MDA5, and ISG15 protein levels in control (*GFP* sgRNAs) and STING-deficient HCC366 cells (n=2). β-Actin was used as a loading control. (**c**) Control and STING-deficient HCC366 cells were transduced with lentivirus expressing Cas9 and sgRNAs targeting *GFP* or *ADAR*. Cell viability was assessed 13 days after lentiviral transduction by crystal violet staining. A representative image of crystal violet staining (left) and quantitation of cell viability (right) from one experiment is shown. Cell viability values were normalized to the GFP sg2 control #2 within each group of isogenic cell lines.

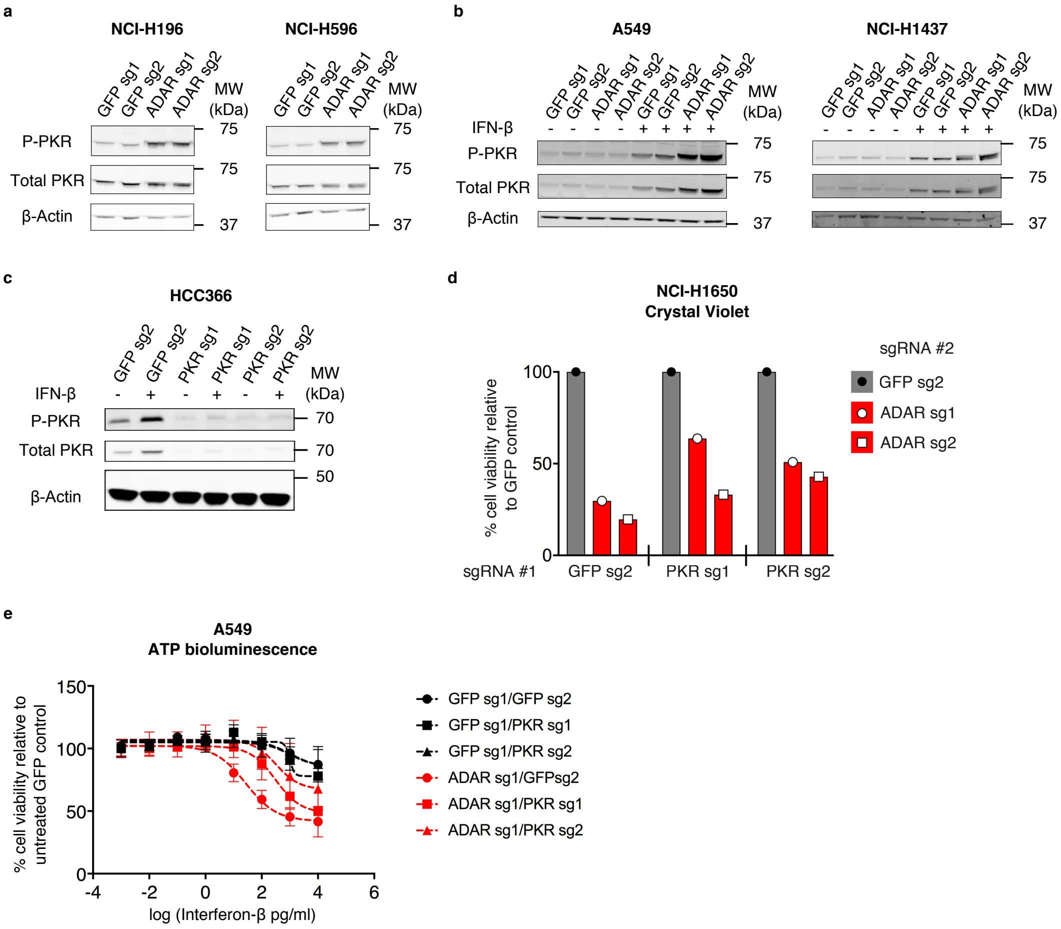

Supplementary Figure 5. Cell lethality after ADAR1 loss is partially mediated through activation of PKR signaling. This figure is related to Figure 3. (**a**) Immunoblots showing protein levels of phosphorylated and total PKR 5 days after *GFP* or *ADAR* deletion by CRISPR-Cas9 in the indicated *ADAR* KO-sensitive cell lines (n=2 for each cell line). β-Actin served as a loading control. (**b**) Immunoblots showing protein levels of phosphorylated and total PKR in ADAR1-deficient A549 and NCI-H1437 cells 24 hours after treatment with vehicle or IFN-β (10 ng/mL) (n=2). β-Actin served as a loading control. (**c**) Validation of PKR deletion in HCC366 cells by immunoblotting after gene KO with CRISPR-Cas9. Protein lysates were harvested after 24 hours of treatment with vehicle or IFN-β (10 ng/mL) and were probed with antibodies against phospho-PKR, total PKR, and β-Actin (loading control) (n=2). (**d**) Cell viability of control (GFP sg2) or PKR-deficient NCI-H1650 cells was assessed by crystal violet staining 8 days after *GFP* or *ADAR* KO with CRISPR-Cas9. Quantitation of cell viability (right) is shown (n=1). Cell viability values were normalized to the GFP sg2 control #2 within each group of isogenic cell lines. (**e**) Cell viability of control, PKR-deficient, ADAR1-deficient, or ADAR1/PKR double-deficient A549 cells was assessed by ATP bioluminescence 3 days after treatment with vehicle or IFN-β (10 ng/mL). ATP bioluminescence values were normalized to the vehicle-treated control within each isogenic cell line. Data from two biological replicates are shown. **Note:** ADAR sg1 was used in this experiment. Error bars represent standard deviation in all graphs.

Supplementary Figure 6

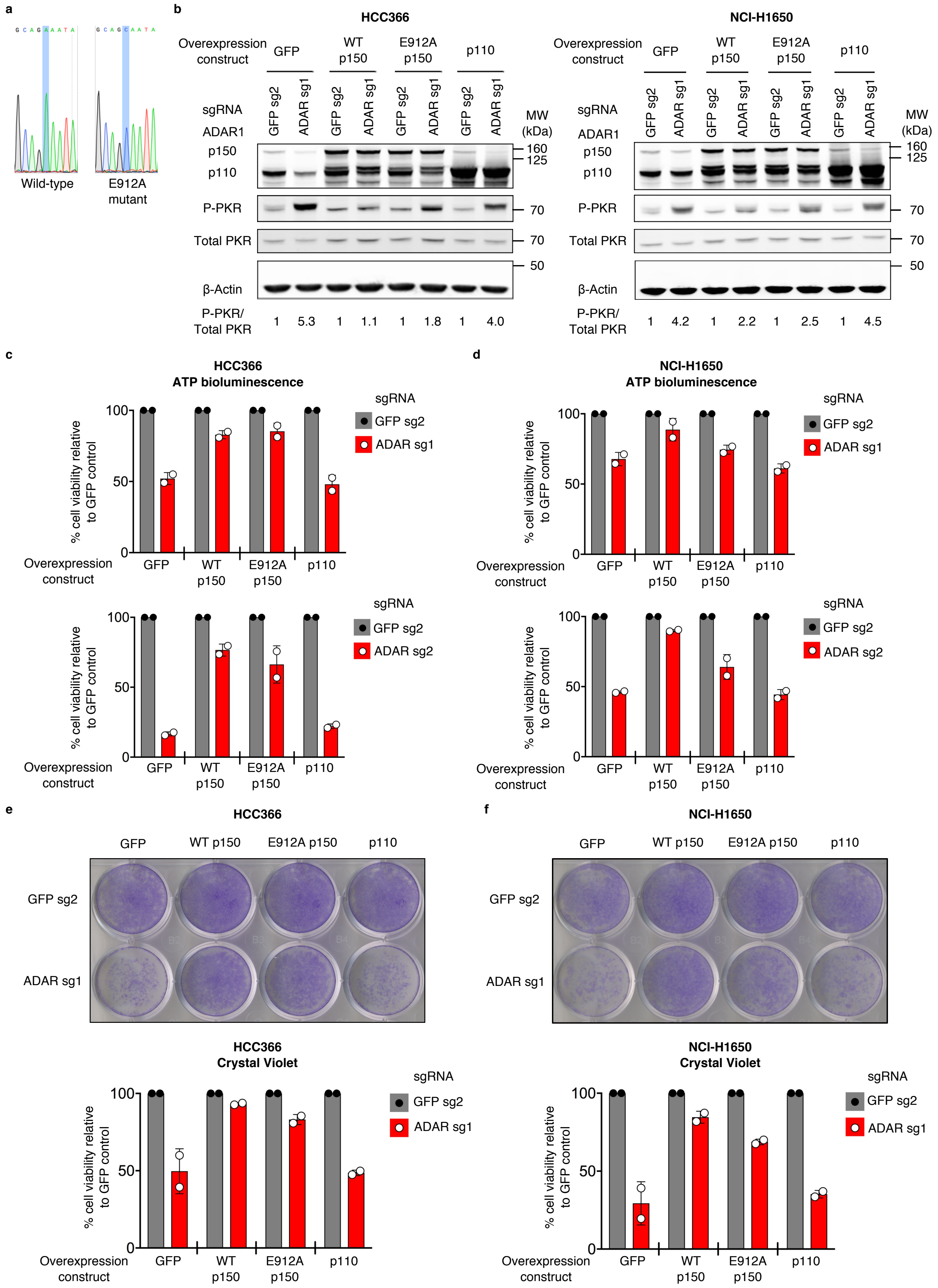

Supplementary Figure 6. Both non-enzymatic and catalytic functions of ADAR1-p150 may be important to prevent cell lethality in cancer cell lines. This figure is related to Figure 4. In this figure, panels (**b**), (**e**), and (**f**) present similar data as panels (**a**), (**b**), and (**c**) in Figure 4, respectively, but utilize a different sgRNA targeting ADAR (sg1). (**a**) Generation of the E912A ADAR1-p150 mutant using site-directed mutagenesis. (**b)** GFP control, WT ADAR1-p150, E912A ADAR1-p150, or WT ADAR1-p110 proteins were overexpressed in HCC366 (right) or NCI-H1650 cells (left) prior to transduction with lentivirus that co-expressed Cas9 and sgRNAs targeting *GFP* (control) or *ADAR* (**using ADAR sg1**). Protein lysates were collected 6 days after *GFP* or *ADAR* KO and probed with antibodies against ADAR1, phospho-PKR, total PKR, and β-Actin (loading control) in HCC366 (left) or NCI-H1650 cells (right). The fold change in the phospho-PKR to total PKR ratio (P-PKR/Total PKR) relative to the corresponding GFP sgRNA control is shown in each lane (n=2). (**c, d**) Cell viability of control or ADAR1-overexpressing HCC366 (**c**) or NCI-H1650 (**d**) cells was assessed by ATP bioluminescence 9 days after *GFP* or *ADAR* KO with CRISPR-Cas9. Top graphs show data obtained using **ADAR sg1** and bottom graphs shown data obtained using **ADAR sg2** (n=2 for both cell lines). Cell viability values were normalized to the GFP sg2 control within each pair of isogenic cell lines. (**e, f**) Cell viability of GFP-overexpressing (control) or ADAR1-overexpressing HCC366 (**e**) or NCI-H1650 (**f**) cells was assessed by crystal violet staining 11 to 16 days after *GFP* or *ADAR* KO with lentivirus co-expressing Cas9 and **ADAR sg1**. A representative image of crystal violet staining (top) and quantitation of cell viability (bottom) from two independent biological replicates for each cell line are shown. Cell viability values were normalized to the GFP sg2 control within each pair of isogenic cell lines. Error bars represent standard deviation in all graphs.

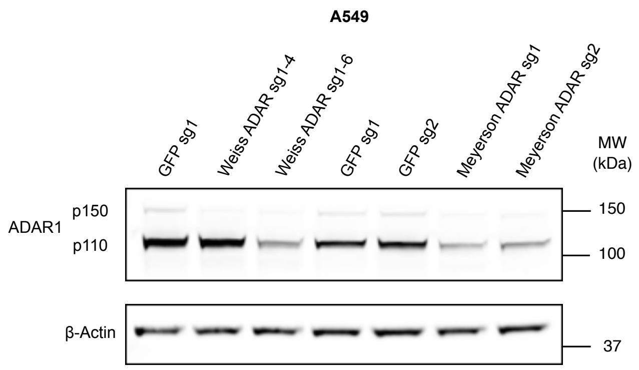

Supplementary Figure 7. Knockdown of ADAR1 protein in A549 cells after CRISPR-Cas9-mediated *ADAR* KO. A549 cells obtained from the CCLE were transduced with lentivirus co-expressing Cas9 and sgRNAs targeting *GFP* (control) or *ADAR*. Weiss ADAR sg1-4 and Weiss ADAR sg1-6 sgRNA sequences were generated based on the study by Li and Banerjee et al.[1], while Meyerson ADAR sg1 and Meyerson ADAR sg2 sgRNA sequences are shown in Supplementary Table 2. Transduced A549 cells were selected with puromycin (2 μg/mL) for 10 days, at which point protein lysates were collected. Immunoblotting was performed with antibodies against ADAR1 and β-Actin (loading control) (n=1).

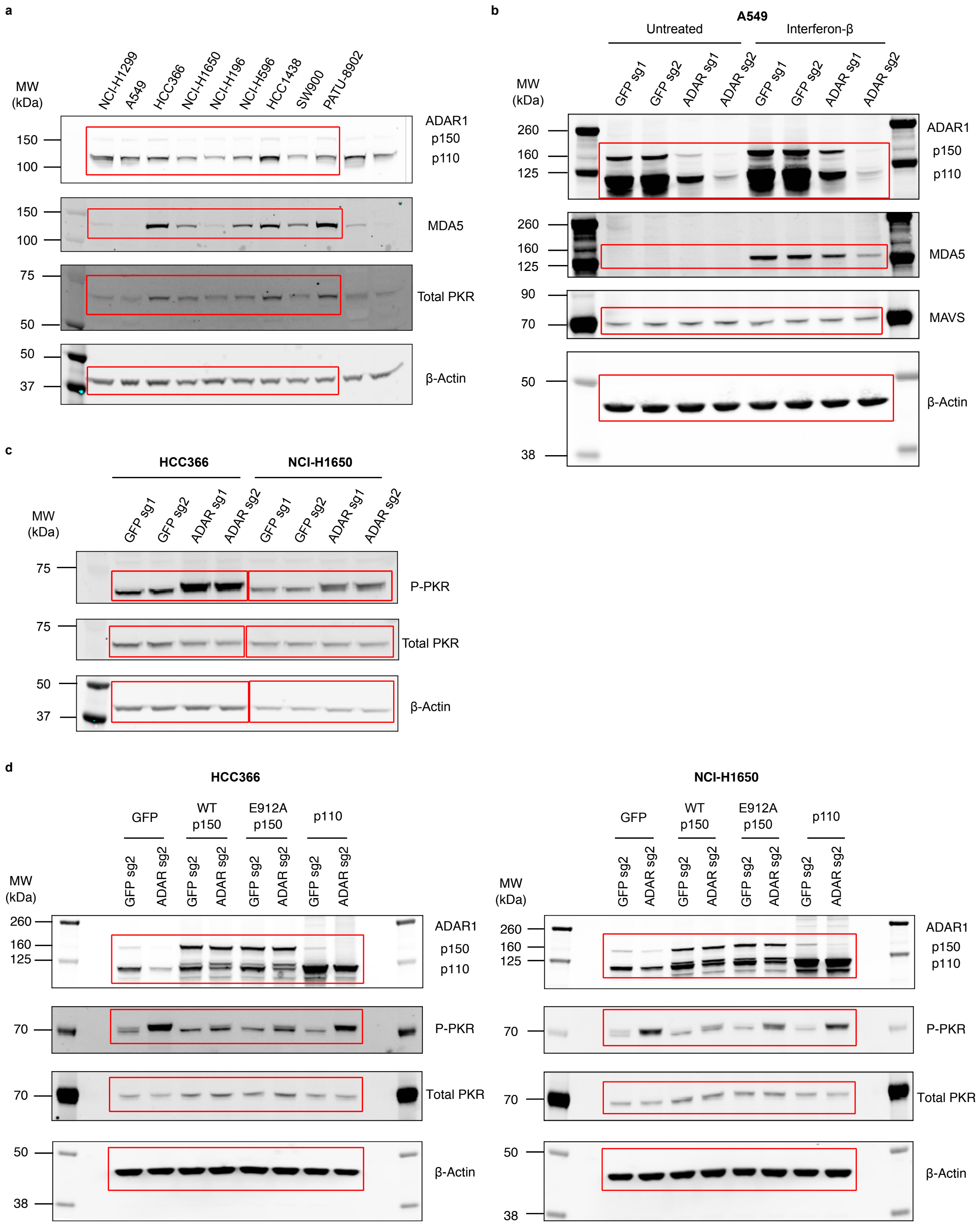

Supplementary Figure 8. Uncropped immunoblots. Uncropped versions of the cropped immunoblots shown in Figure 1c (**a**), Figure 2d (**b**), Figure 3b (**c**), and Figure 4a (**d**) are shown. The red boxes outline the approximate areas that were cropped out.

Supplementary Tables

Gene	Number of six standard deviation cell lines	Minimum dependency z-score	98k library lines	55k library lines	Non-lung six standard deviation count	Lung six standard deviation count	Remaining non-lung count	Remaining lung count	Chi-squared test p value	Fisher exact test p value
SMARCA2	9	-9.961	285	216	0	9	385	107	0.0000	0.0000
ATP5H	3	-8.123	285	216	0	3	385	113	0.0016	0.0122
PRKDC	3	-7.137	285	216	0	3	385	113	0.0016	0.0122
ADAR	2	-6.099	285	216	0	2	385	114	0.0098	0.0533
CSDE1	2	-8.606	285	216	0	2	385	114	0.0098	0.0533
CSNK2B	2	-7.489	285	216	0	2	385	114	0.0098	0.0533
CXCR4	2	-7.339	285	216	0	2	385	114	0.0098	0.0533
FBXL5	2	-7.315	285	216	0	2	385	114	0.0098	0.0533
RACGAP1	2	-7.632	285	216	0	2	385	114	0.0098	0.0533
SARS	2	-6.867	285	216	0	2	385	114	0.0098	0.0533
USPL1	2	-7.022	285	216	0	2	385	114	0.0098	0.0533

Supplementary Table 1. Genes displaying outlier lethality in lung cancer cells included in published genome-scale shRNA loss-of-function screens[2].

Protein	Guide	Target sequence
GFP	GFP sg1	GGAGCGCACCATCTTCTTCA
GFP	GFP sg2	GAAGTTCGAGGGCGACACCC
NA	Non-targeting	GCTTGAGTGTATGCACAAAT
ADAR1	ADAR sg1	GTGCATACACTCAAGCAGTG
ADAR1	ADAR sg2	AGATAGCCATGCTGAGCCAC
IFNAR1	IFNAR1 sg1	GATCTAATGTTAAAGACTGG
IFNAR1	IFNAR1 sg2	GAACAAAAGATAGTGTTATG
MDA5	MDA5 sg1	GTGCATATGCGCTTTCCCAG
MDA5	MDA5 sg2	AGGACTGAGGAATCAGCACG
MAVS	MAVS sg1	CCTCTCCTGGAACTTCCGGT
MAVS	MAVS sg2	GGTATTGAAGAGATGCCAGA
PKR	PKR sg1	GCAAGACTATGGAAAGGAAG
PKR	PKR sg2	AAAGGCAATACGTACCACTG

Supplementary Table 2. sgRNA sequences utilized for CRISPR-Cas9-mediated gene deletion.

Supplementary Data Files 1 through 3 are provided separately as Microsoft Excel files.

Supplementary References

1. Li, Y.*, et al.* Ribonuclease L mediates the cell-lethal phenotype of double-stranded RNA editing enzyme ADAR1 deficiency in a human cell line. *Elife* **6**(2017).
2. Tsherniak, A.*, et al.* Defining a Cancer Dependency Map. *Cell* **170**, 564-576 e516 (2017).
