# Supplementary material for: Identification of ADAR1 adenosine deaminase dependency in a subset of cancer cells
Source: Nat Commun. 2018 Dec 21;9:5450. doi: 10.1038/s41467-018-07824-4 (PMC6303303; doi:10.1038/s41467-018-07824-4)
Supplement: Supplementary file 6 — Description of Additional Supplementary Files [file 41467_2018_7824_MOESM6_ESM.pdf]

Supplementary Data File 1. Differential gene expression between *ADAR* KO-sensitive and KO-insensitive lung cancer cell lines. Analysis of differential gene expression between *ADAR* KO-sensitive (annotated as 1 in column B) and KO-insensitive (annotated as 2 in column B) lung cancer cell lines (listed in column A). Individual genes are listed in column D. The mean fold change in gene expression between *ADAR* KO-sensitive and KO-insensitive lung cancer cell lines are shown in column E. *p* values were calculated by the Mann-Whitney U test and are displayed in column F. p values < 0.05 were considered to be statistically significant. Gene expression data were obtained from the CCLE Data Portal [http://www.broadinstitute.org/ccle].

Supplementary Data File 2. Interferon gene expression signature scores for cancer cell lines in the CCLE. To generate interferon gene expression signature scores for CCLE cancer cell lines, a list of 27 ISGs that were significantly differentially expressed between *ADAR* KO-sensitive and KO-insensitive lung cancer cell lines was compiled from Supplementary Data File 1. Individual cancer cell lines are listed in column A. The gene expression levels (measured as reads per kilobase million) of each of the 27 ISGs are listed in columns B through AB. The calculated interferon gene expression signature scores for each cancer cell line are displayed in column AC and the corresponding z-scores and are displayed in column AD. Gene expression data were obtained from the CCLE Data Portal [http://www.broadinstitute.org/ccle].

Supplementary Data File 3. Differential gene expression between an expanded set of *ADAR* KO-sensitive and KO-insensitive cancer cell lines. Analysis of differential gene expression between *ADAR* KO-sensitive (annotated as 1 in column B) and KO-insensitive (annotated as 2 in column B) cancer cell lines of diverse lineages. Individual genes are listed in column D. The log2(mean fold change) in gene expression between *ADAR* KO-sensitive and KO-insensitive cancer cell lines are shown in column E. *p* values were calculated by the Mann-Whitney U test and are displayed in column F. p values < 0.05 were considered to be statistically significant. q values were calculated from the corresponding *p* values and are displayed in column G. The -log(q value) for each gene is shown in column H. Gene expression data were obtained from the CCLE Data Portal [http://www.broadinstitute.org/ccle].
